# Cloning, phylogeny, and expression analysis of the Broad-Complex gene in the longicorn beetle *Psacothea hilaris*

**DOI:** 10.1186/2193-1801-3-539

**Published:** 2014-09-18

**Authors:** Keisuke Nagamine, Takumi Kayukawa, Sugihiko Hoshizaki, Takashi Matsuo, Tetsuro Shinoda, Yukio Ishikawa

**Affiliations:** Graduate School of Agricultural and Life Sciences, The University of Tokyo, Tokyo, 113-8657 Japan; National Institute of Agrobiological Sciences, Tsukuba, 305-8634 Japan

**Keywords:** *Psacothea hilaris*, *Broad-Complex*, Holometabola, Starvation

## Abstract

Seven isoforms of *Broad-Complex* (*PhBR-C*), in which the sequence of the zinc finger domain differed (referred to as Z1, Z2, Z3, Z2/Z3, Z4, Z5/Z6, and Z6, respectively), were cloned from the yellow-spotted longicorn beetle *Psacothea hilaris*. The Z1–Z4 sequences were highly conserved among insect species. The Z5/Z6 isoform was aberrant in that it contained a premature stop codon. Z6 had previously only been detected in a hemimetabola, the German cockroach *Blattella germanica*. The presence of Z6 in *P. hilaris*, and not in other holometabolous model insects such as *Drosophila melanogaster* or *Tribolium castaneum*, suggests that Z6 was lost multiple times in holometabolous insects during the course of evolution. *PhBR-C* expression levels in the brain, salivary gland, and epidermis of larvae grown under different feeding regimens were subsequently investigated. *PhBR-C* expression levels increased in every tissue examined after the gut purge, and high expression levels were observed in prepupae. A low level of *PhBR-C* expression was continuously observed in the brain. An increase was noted in *PhBR-C* expression levels in the epidermis when 4th instar larvae were starved after 4 days of feeding, which induced precocious pupation. No significant changes were observed in expression levels in any tissues of larvae starved immediately after ecdysis into 4th instar, which did not grow and eventually died.

**Electronic supplementary material:**

The online version of this article (doi:10.1186/2193-1801-3-539) contains supplementary material, which is available to authorized users.

## Background

The yellow-spotted longicorn beetle *Psacothea hilaris* (Coleoptera: Cerambycidae) is an important pest of mulberry and fig trees in East Asia (Kusama and Takakuwa 1984), and has recently settled in Europe (Jucker et al. 2006; Lupi et al. 2013). We have been interested in the developmental responses of *P. hilaris* larvae to starvation (Shintani et al. 2003; Munyiri et al. 2003, 2004; Munyiri and Ishikawa 2005a, [b]) because food shortages are a constraint that insects often have to contend with in nature. This beetle was shown to terminate its larval life precociously and form small pupae in response to the sudden exhaustion of a food supply (Shintani et al. 2003). The larval development of *P. hilaris* is characteristic in that the last instar is not fixed under fully fed conditions (Watari et al. 2002). We previously reported that approximately 50% of 4th-instar *P. hilaris* larvae at 25°C and under a long-day photoperiod molted to the 5th instar on day 13 and pupated approximately 18 days thereafter; the remainder pupated without a larval molt with a mean 4th-instar period of 24 days (Shintani et al. 1996). The starvation of 4th-instar larvae not only strongly suppressed molting to the 5th instar, but also induced precocious (approximately 5 days earlier than normal) pupation in larvae exceeding a threshold weight (180 mg), whereas larvae weighing less than the threshold did not pupate and eventually died (Munyiri et al. 2003). Hemolymph juvenile hormone (JH) levels and ecdysteroid titers in starved and normally fed larvae suggested that starvation induced a rapid decline in the JH titer, and this appeared to cue the early occurrence of a small ecdysteroid peak that committed larvae to metamorphosis (Munyiri and Ishikawa 2005a).

The Broad-Complex gene (*BR-C*) encodes a family of early transcription factors (BR-C isoforms) that are involved in ecdysteroid-signaling pathways in insects. These transcription factors play key roles in specifying the pupal stage in holometabola (Suzuki et al. 2008; Konopova et al. 2011), but are involved in embryogenesis, the growth of wing buds, and wing vein formation in hemimetabola (Erezyilmaz et al. 2006; Piulachs et al. 2010; Huang et al. 2013). All BR-C isoforms have a well-conserved Broad-Tramtrack-Bric-a-brac (BTB) domain in the N-terminus and a variable C_2_H_2_-type zinc finger DNA binding domain in the C-terminus (DiBello et al. 1991). Six types of zinc finger sequences (Z1–Z6) have been identified by phylogenetic analyses (Spokony and Restifo 2007; Piulachs et al. 2010). The combination of zinc finger types used is known to differ depending on the species.

In coleopteran species, starvation-induced precocious pupation similar to that in *P. hilaris* has only been reported for *Dacne picta*, *Onthophagus taurus*, and *Epicauta gorhami* (Sato and Suzuki 2001; Shafiei et al. 2001; Terao et al. 2014). The larvae of these species have been shown to pupate earlier than usual when food resources are limited; however, the mechanisms that lead to early metamorphosis in coleopteran species have not been investigated. In the present study, we examined the expression of *BR-C* under starved conditions to obtain clues to the mechanism underlying the induction of precocious metamorphosis in starved *P. hilaris*. We first cloned all *BR-C* zinc finger isoforms expressed in *P. hilaris* (*PhBR-C*) because no information was available on the sequence of *BR-C* in this beetle. Since this is the first study to obtain the Z6 isoform from a holometabolous insect, the evolution of *BR-C* was discussed with reference to the results of a phylogenetic analysis of zinc finger sequences. We then quantified the transcripts of *BR-C* in larvae growing under different feeding/starvation regimens in order to clarify changes in its expression profile upon food deprivation.

## Materials and methods

### Insects

The insects used in the present study were the offspring of west-Japan type *Psacothea hilaris* collected at Fukuchiyama city in 2005. *P. hilaris* were reared at 25°C, 30–60% relative humidity, and under a photoperiod of 15 h light: 9 h dark. To obtain eggs, a pair of mature adults (2 to 3 weeks old) was placed in a plastic cup (12 cm in diameter × 10 cm in depth) and fed an artificial diet, Insecta LF™ (Nosan Corp. Yokohama, Japan). A cut branch from a mulberry tree (1–2 cm in diameter and 5–7 cm in length) was provided for oviposition. The eggs laid under the cortex of the branch were collected one day later. The eggs were maintained on moistened filter paper in a Petri dish (5.5 cm in diameter × 1.5 cm in depth) with a note of the date of collection. Larvae that hatched within 7 days were transferred singly to new Petri dishes with a piece of the artificial diet to avoid cannibalism. Silkmate 2S™ (Nosan Corp.) was given as the artificial diet until the 3rd instar, and the diet was then switched to Insecta LF™.

### RNA extraction

Tissues were dissected from the larvae of *P. hilaris* in phosphate-buffered saline (PBS, 2.5 mM KCl, 141 mM NaCl, 8.1 mM Na_2_HPO_4_, and 2.5 mM KH_2_PO_4_, pH 7.0) under a stereomicroscope and were then washed in PBS. Groups of brains from 20 larvae, salivary glands from 5 larvae, and integuments from 5 larvae were snap frozen in liquid nitrogen, and stored at -80°C prior to use. Total RNA was isolated from each tissue using the RNeasy Mini Kit (Qiagen, Tokyo, Japan) with RNase-free DNase I (Qiagen) treatment.

### Cloning of the BTB domain

A fragment of the BTB domain of *BR-C* was amplified by degenerate polymerase chain reactions (PCR) according to Zollman et al*.* (1994). PCR was conducted in a 20-μl reaction volume with 1 μl genomic DNA as a template, TaKaRa Ex *Taq* 1.25 U, dNTP mixture 0.5 μM, 1 × Ex Taq buffer (Takara), and 5 μM primers (Br-f1/Br-R1 or Br-f1/Br-R2; Additional file 1: Table S1). Thermal cycle conditions were 95°C for 5 min, followed by 3 cycles of 95°C for 1 min, 40°C for 2 min, and 72°C for 2 min, 30 cycles of 95°C for 1 min, 55°C for 2 min, and 72°C for 2 min, and a final extension of 72°C for 5 min. The product was cloned using pGEM-T Easy Vector System I (Promega), and sequenced using the BigDye Terminator v1.1 Cycle Sequencing Kit (Applied Biosystems).

### Determination of 3′-end and 5′-end sequences

The 3′- and 5′-end sequences of *BR-C* were obtained by 3′- and 5′-RACE methods using the CapFishing Full-length cDNA Premix Kit (Seegene, Seoul, Korea). First strand cDNA was synthesized from total RNA extracted from the brain–corpora cardiaca–corpora allata complexes of four prepupae and was used as the template. Primers were designed based on the sequence of the gene fragment obtained by PCR using degenerate primers (Additional file 1: Table S1).

### Sequencing of Z2, Z3, Z2/Z3, and Z5 zinc finger domains

To determine the sequences of the Z2 and Z3 zinc finger sequences, the reverse degenerate primers, Z2-DG1 and Z3-DG1 were designed based on the sequences of the region well conserved among the Z2 and Z3 isoforms (Additional file 1: Table S1). BR-FW2 was used as the forward primer. First strand cDNA was synthesized from total RNA isolated from the salivary glands of prepupa with an oligo-dT adaptor primer using the PrimeScript 1st strand cDNA Synthesis kit (Takara Bio). The conditions for PCR were: 94°C for 3 min followed by 40 cycles of 94°C for 30 sec, 40°C for 30 sec, and 72°C for 1 min with an annealing temperature that decreased by 0.5°C for each cycle until it reached 30°C, and a final incubation at 72°C for 3 min. To determine the sequence of the Z5 zinc finger domain, the reverse degenerate primers, Z5-DGRV1 and Z5-DGRV2 were designed for the first and nested PCRs, respectively, based on the sequences of the region well conserved among the Z5 isoform (Additional file 1: Table S1). BR-FW2 was used as the forward primer. First strand cDNA was synthesized from total RNA isolated from the brains for the templates of the first PCR. The products of the first PCR were diluted 1,000-fold, and then used as templates for nested PCR. The same conditions were used for the first and nested PCRs: 94°C for 3 min followed by 5 cycles of 94°C for 30 sec and 72°C for 5 min; 5 cycles of 94°C for 30 sec and 68°C for 5 min; 5 cycles of 94°C for 30 sec, 65°C for 30 sec, and 68°C for 5 min; 5 cycles of 94°C for 30 sec, 62°C for 30 sec, and 68°C for 5 min; 5 cycles of 94°C for 30 sec, 58°C for 30 sec, and 68°C for 5 min; and a final incubation at 72°C for 3 min. The primers Z2-FW1, Z2-FW2, Z2-FW3, Z3-FW1, Z3-FW2, Z5-PWFW1, and Z5-PWFW2 were used in 3′-RACE to determine the 3′-end sequences of the Z2, Z2/Z3, Z3, and Z5/Z6 isoforms (Additional file 1: Table S1).

### Search of homologs and phylogenetic analyses

The homologs of BR-C were searched for using tblastx (http://www.ncbi.nlm.nih.gov/) with the deduced amino acid sequence of *PhBR-C* as the query. Regarding a phylogenetic analysis of the zinc finger domains, 98 zinc finger sequences obtained from public databases were preliminarily aligned by Clustal W (Thompson et al. 1994). Sequences with many gaps were subsequently excluded from further analyses. Regarding *Drosophila* species, only the sequences of *D. melanogaster* were retained as representatives of the genus. Identical sequences shared by different species, screened using MacClade ver.4 (Maddison and Maddison 2005), were finally grouped as an operational taxonomic unit (OTU).

Forty-five sequences of 52 amino acid residues, among which the Z5 of *A. mellifera* had gaps, were used in the phylogenetic analysis (Additional file 2: Table S2). The JTT + G model was selected as a best-fit model of amino acid substitutions based on the Bayesian information criterion by MEGA5 (Tamura et al. 2011). The phylogenetic relationship was reconstructed by the maximum likelihood method using MEGA5, and the bootstrap test was performed with 100 resamplings.

### Experimental feeding regimens

**Figure 1:**
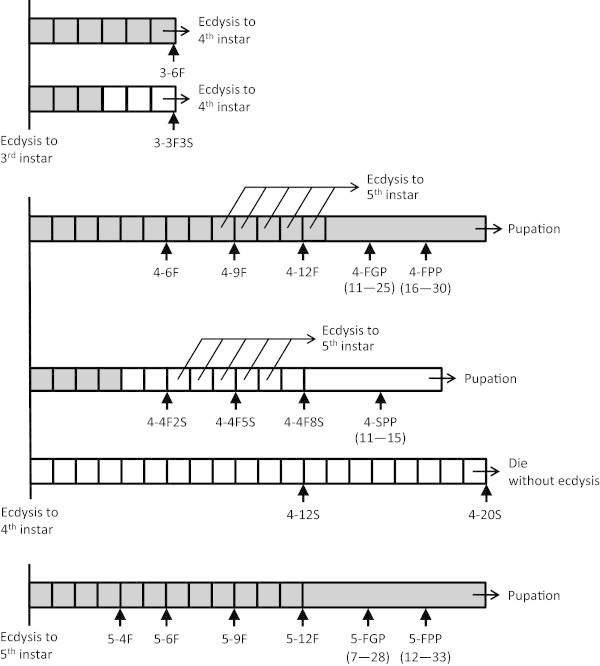
**Schematic representations of the feeding and starvation regimens.** Filled and open boxes indicate fed and starved conditions, respectively. Arrows directed upward indicate the sampling time . Completion of the gut purge (4-FGP and 5-FGP) was confirmed by direct observations through the integument. The prepupal state (4-FPP, 4-SPP, and 5-FPP) was diagnosed by apolysis of the integument and the refractoriness of insects when stimulated with tweezers. Pupation occurred approximately 10 days after the gut purge (4-FGP and 5-FGP) and 5 days after becoming prepupa (4-FPP, 4-SPP, and 5-FPP). Numerals in parentheses indicate days after ecdysis.

In this study, the feeding/starvation regimen was denoted by, for example, 4-4F8S, in which the first number indicated the instar of the larva and that before F or S indicated the length (days) of feeding (F) or starvation (S). Accordingly, 4-4F8S indicated that from the day of ecdysis into 4th instar, larvae were fed for 4 days and then starved for 8 days before being sampled. Insects were sampled at specified times, as shown in Figure 1, and dissected to isolate tissues. Molting of insects was checked every 6 h when necessary. In the samplings of 3-3F and 3-3S (Figure 1), most individuals were ready for ecdysis, and those that had already ecdysed were excluded. Since some of the 4th-instar larvae ecdysed into 5th instar on day 11 under normally fed conditions, 4-9F and 4-12F samples may have contained larvae ready for the next larval molt and those preparing for pupal molt. Larvae that were deprived of food right after ecdysis into 4th instar did not molt into 5th instar or pupate, and eventually died.

### Quantitative real-time PCR (qPCR)

Transcripts of the core region of *PhBR-C* were quantified by qPCR using an ABI PRISM 7700 genetic analyzer (Applied Biosystems). Total RNA prepared from each tissue was reverse transcribed using the PrimeScript RT reagent Kit (Takara-bio) with random 6mers as primers. The reaction was conducted in a 20-μl volume, and the product was used as the cDNA template. To quantify *PhBR-C*, the reverse transcripts were diluted 100-fold, and BRcore-FW2, and BRcore-RV2 were used as primers (Additional file 1: Table S1). *PhBR-C* from the salivary glands of 4-FPP insects was diluted 10–100,000-fold and used as standards. To quantify *18S rRNA*, a reference gene, first strand cDNA, from each sample was diluted 1,000-fold, and PCR was conducted with 18S-FW1 and 18S-RV1 as primers (Additional file 1: Table S1). *18S rRNA* from the midguts of 5-FPP insects was diluted 100–1,000,000-fold and used as standards. PCR was conducted in a 20-μl reaction volume with the template, 2 μl of 1 × SYBR *Premix Ex Taq*, 2 μl of 1 × ROX Reference Dye (Takara-bio), and 0.2 μM of primers. PCR conditions were: 95°C for 10 sec followed by 40 cycles of 95°C for 5 sec and 60°C for 30 sec. The amount of *PhBR-C* RNA was normalized to that of *18S rRNA*. Quantification was repeated 3 times.

## Results

### *PhBR-C*cDNA

**Figure 2:**
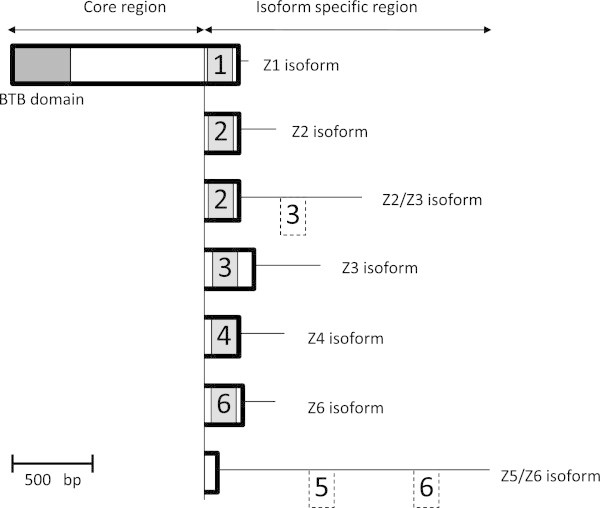
**Schematic representation of the structures of the** ***PhBR-C*** **transcripts found in the present study.** The dark and light gray boxes indicate the BTB domain and zinc finger sequences, respectively. The thin line and dotted box indicate the 3′ UTR and zinc finger sequence in 3′ UTR, respectively. The number in the box represents the type of zinc finger sequence. The variation in 5′ UTR was not investigated in the present study. The GenBank accession numbers of Z1, Z2, Z2/Z3, Z3, Z4, Z6, and Z5/Z6 transcripts were AB857715, AB857716, AB858990, AB858991, AB858992, AB858994, and AB858993, respectively.

**Figure 3:**
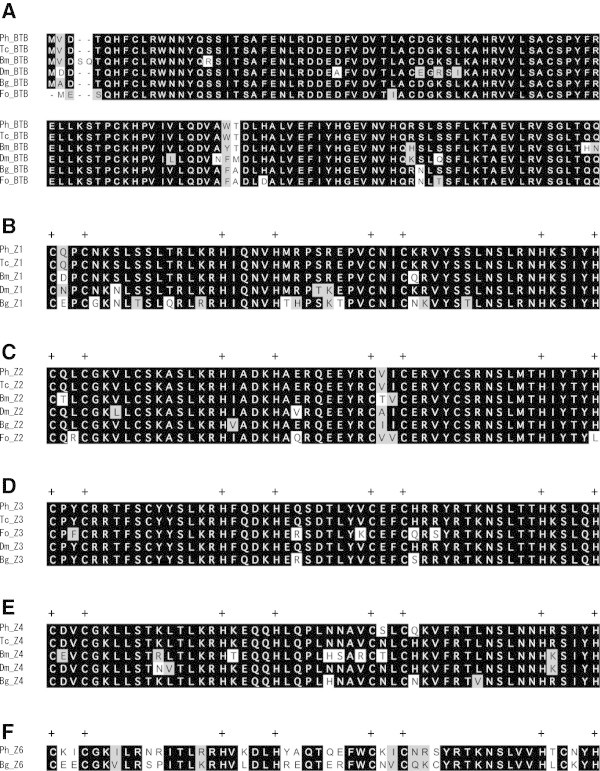
**Comparison of the predicted amino acid sequences of BR-Cs.** Ph: *Psacothea hilaris*. Tc: *Tribolium castaneum*. Dm: *Drosophila melanogaster*. Bm: *Bombyx mori*. Bg: *Blattella germanica*. Fo: *Frankliniella occidentalis*. The sequences were aligned for BTB **(A)**, Z1 **(B)**, Z2 **(C)**, Z3 **(D)**, Z4 **(E)**, and Z6 **(F)**. Plus signs (+) indicate conserved Cys and His residues. Black and light gray shading indicate identical and similar amino acid residues, respectively.

**Figure 4:**
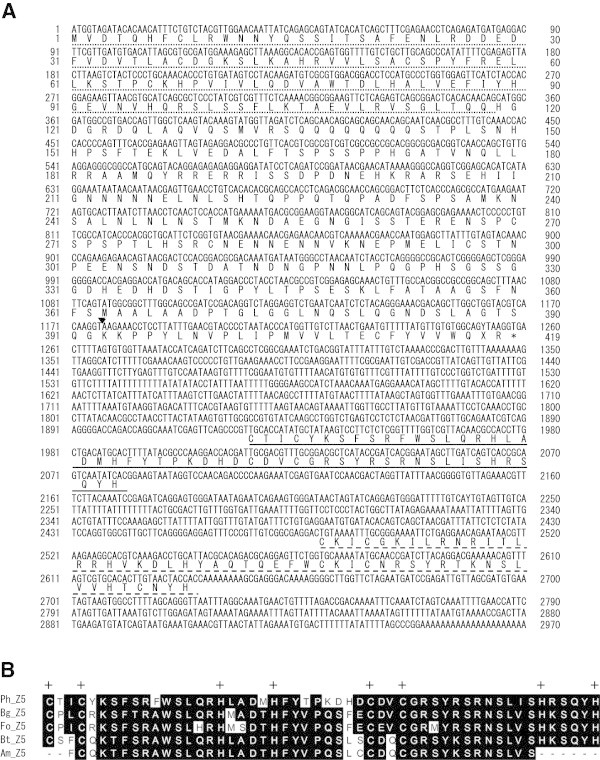
**The Z5/Z6 isoform. (A)** Nucleotide and deduced amino acid sequences of the Z5/Z6 isoform. The BTB domain is indicated by a dotted underline. The primer positions for qPCR are indicated by lowercase letters. The triangle between positions 1176 and 1177 indicates the end of the core region. An asterisk indicates the stop codon (TGA). Sequences homologous to Z5 and Z6 in 3′ UTR are indicated by an underline and dashed underline, respectively. **(B)** Comparison of the deduced amino acid sequence of Z5 from *Psacothea hilaris* (Ph) with those of Z5 from *Blattella germanica* (Bg), *Frankliniella occidentalis* (Fo), *Bombus terrestris* (Bt), and *Apis mellifera* (Ap). Plus signs (+) indicate the conserved Cys and His residues of zinc fingers. Black shading indicates identical amino acid residues.

Genomic DNA was prepared from the fat body of *P. hilaris* larvae. PCR using degenerate primers was performed to amplify fragments (ca. 100-bp) of the BTB domain of *BR-C*. Among the 34 sequences cloned, four were identical and showed a high degree of similarity to the *BR-C* of *D. melanogaster* (*DmBR-C*)*, M. sexta* (*MsBR-C*), *B. mori* (*BmBR-C*), and *Aedes aegypti* (*AaBR-C*). We named the gene containing this sequence *PhBR-C*. 3′- and 5′-RACE experiments using cDNA prepared from the brain–corpora cardiaca–corpora allata complexes of *P. hilaris* revealed three types of sequences, which differed in the zinc finger domain, in the transcripts of *PhBR-C*. The *BR-C* core region including the BTB domain was common among the three sequences. An analysis of the three zinc finger sequences with blastx showed that they were homologous to the Z1, Z4, and Z6 sequences of *BR-C* in other insects (Figures 2 and 3). This is the first study to obtain the Z6 isoform from a holometabolous insect.To clone the Z2, Z3, and Z5 isoforms that were not found in the preceding 3′-RACE experiments, PCR was conducted using the cDNA template prepared from the salivary glands or brains of 5-FPP insects. Degenerate reverse primers were designed based on amino acid sequences in the zinc finger domain of the Z2, Z3, and Z5 isoforms available from databases, and a forward primer was designed based on the sequence of the core region. Accordingly, sequences were obtained that were considered to be part of the Z2, Z3, and Z5 isoforms. 3′-RACE experiments using these sequences revealed the ORFs and 3′UTRs of four different sequences. We named them the Z2, Z3, Z2/Z3, and Z5/Z6 isoforms based on the zinc finger sequences they contained (Figures 2, 3 and 4).

### Comparison of BTB and zinc finger domains

The deduced amino acid sequence of BTB and that of the zinc finger domain in each variant (Z1–Z6) were highly conserved among different orders of insects including both holometabola and hemimetabola (Figures 3 and 4B). The Cys and His residues, which are characteristic of the C_2_H_2_-type zinc finger, were completely conserved (Figures 3 and 4B).

### Structures of the Z2/Z3 and Z5/Z6 isoforms

In addition to the standard Z2 isoform, an isoform that contained a sequence homologous to Z3 in its 3′UTR in addition to the Z2 zinc finger in the ORF was discovered (Z2/Z3 isoform). Furthermore, in addition to the standard Z6 isoform, an isoform was obtained that contained two sequences homologous to Z5 and Z6 isoforms in this order in its 3′UTR (Z5/Z6 isoform; Figures 2 and 4A). A protein that lacks a zinc finger domain is considered to be produced from this mRNA. No BR-C isoform with an intact Z5 zinc finger has been discovered in *P. hilaris*.

### Phylogenetic relationships of zinc finger variants

**Figure 5:**
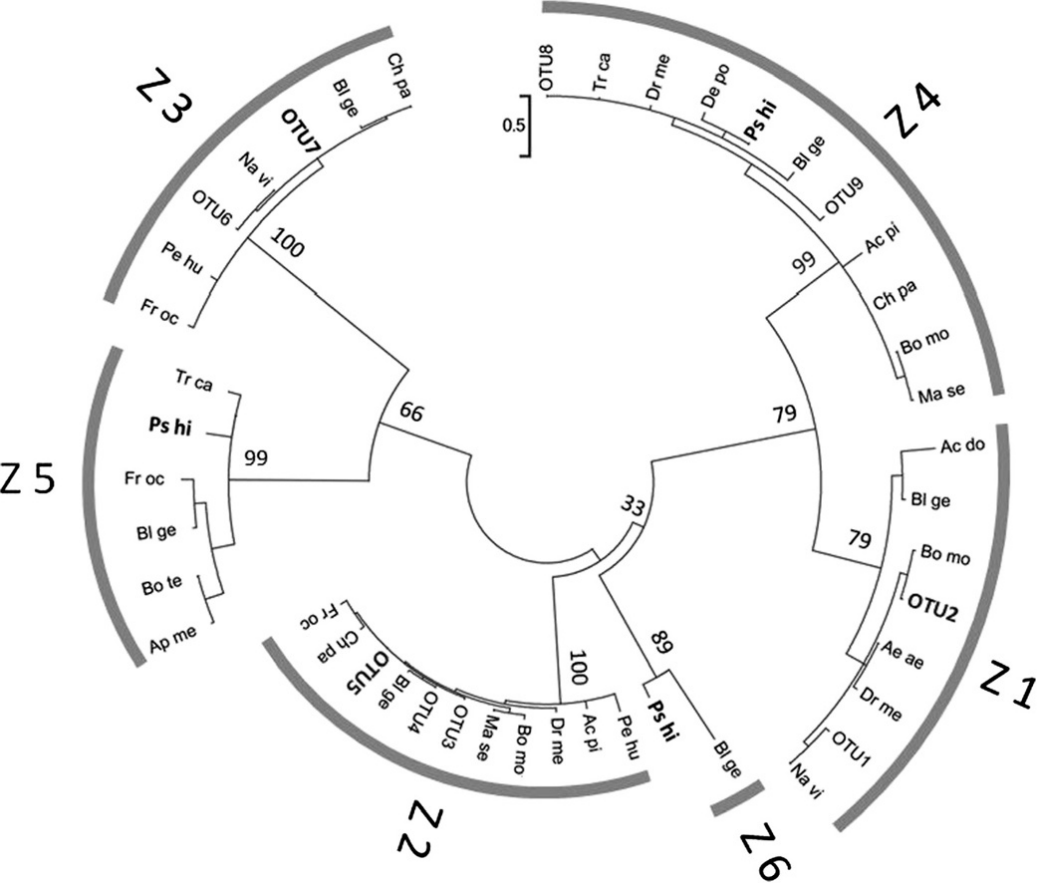
**The maximum likelihood phylogenetic tree of BR-C from various insects.** Bootstrap support indices are shown above major branches. *Psacothea hilaris* and OTUs including *P. hilaris* are indicated by bold letters. Ac do: *Acheta domesticus.* Ac pi: *Acyrthosiphon pisum.* Ae ae: *Aedes aegypti.* Ap me: *Apis mellifera.* Bl ge: *Blattella germanica.* Bo mo: *Bombyx mori.* Bo te: *Bombus terrestris.* De po: *Dendroctonus ponderosae.* Fr oc: *Frankliniella occidentalis.* Ps hi: *Psacothea hilaris.* Tr ca: *Tribolium castaneum.* Na vi: *Nasonia vitripennis.* Dr me: *Drosophila melanogaster.* Ma se: *Manduca sexta.* Pe hu: *Pediculus humanus corporis.* Ch pa: *Chrysopa pallens.* OTU1: *Apis florea, A. mellifera,* and *B. terrestris.* OTU2: *D. ponderosae, P. hilaris,* and *T. castaneum*. OTU3: *A. aegypti, Anopheles gambiae,* and *Culex quinquefasciatus*. OTU4: *A. florea, A. mellifera, Bombus impatiens, B. terrestris,* and *N. vitripennis*. OTU5: *D. ponderosae, P. hilaris,* and *T. castaneum.* OTU6: *A. florea, A. mellifera, B. impatiens, B. terrestris,* and *Megachile rotundata*. OTU7: *A. aegypti, A. gambiae, Bactrocera dorsalis, B. mori, C. quinquefasciatus, D. ponderosae, D. melanogaster, P. hilaris,* and *T. castaneum.* OTU8: *A. aegypti* and *A. gambiae.* OTU9: *A. florea* and *A. mellifera*. The GenBank accession numbers of the sequences used are listed in Additional file 2: Table S2.

The homologs of Z1–Z6 were extensively searched for in public databases using tblastx with the deduced amino acid sequences of *PhBR-C* Z1–Z6 as the query. The Z1–Z5 sequences were respectively obtained from various orders of insects including holometabola and hemimetabola. Z6 was only found in a single species, *Blattella germanica*. The unrooted phylogenetic tree of the Z1–Z6 sequences in various insects (Figure 5) showed that the Z1–Z6 groups were respectively monophyletic. Z1 and Z4 together formed a well-supported clade. We confirmed that the zinc finger sequences found in the *PhBR-C* transcripts were correctly classified into Z1–Z6 types.

### *PhBR-C*transcript levels

**Figure 6:**
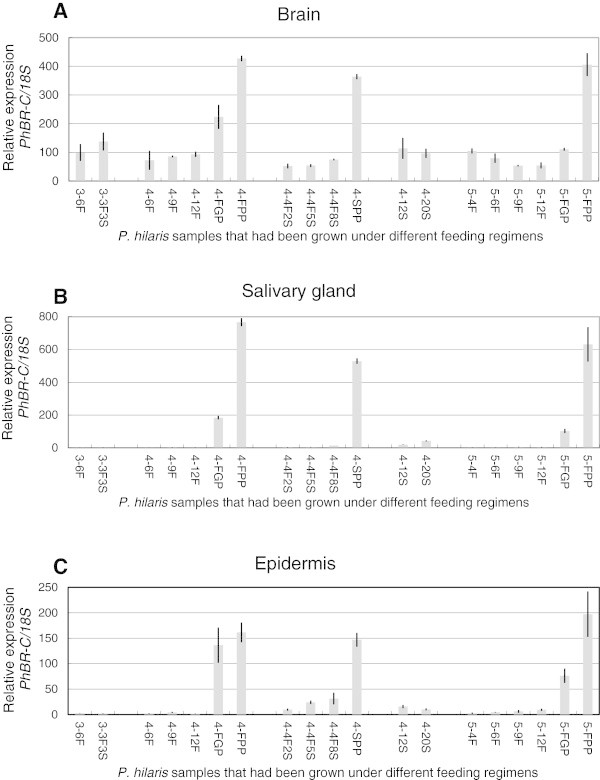
**qPCR analysis of** ***PhBR-C*** **mRNA levels in the brain (A), salivary gland (B), and epidermis (C).** *PhBR-C* mRNA levels (core region) were normalized to those of the internal standard, *18S* ribosomal RNA. *P. hilaris* larvae were grown under different feeding/starvation regimens and sampled at specified times, as shown in Figure 1. Note that 4-12F includes individuals that will soon molt into 5th instar as well as those that will pupate after extended larval growth. The error bar indicates standard deviation (n=3).

Insects were reared under different feeding/starvation regimens, as shown in Figure 1. *PhBR-C* levels in the brain, salivary gland, and epidermis of larvae grown under different feeding regimens (Figure 1) were determined by quantitative PCR using primers specific to the core region (Figure 6). An increase in *PhBR-C* levels was observed in the salivary gland and epidermis of continuously fed larvae after the gut purge (4-FGP and 5-FGP), and further increases in *PhBR-C* levels were observed in prepupae (4-FPP and 5-FPP). No increase was observed in *PhBR-C* levels in 3-6F or 3-3F3S larvae, which were expected to molt soon into 4th instar. No increase was also observed in 4-12F larvae, which included individuals expected to molt soon into 5th instar as well as those expected to pupate after an extended larval period. An increase in *PhBR-C* levels was found in the epidermis when 4th instar larvae were starved after 4 days of feeding (4-4F5S), which induced precocious pupation. Meanwhile, no marked change in expression levels was observed in any tissues of larvae starved immediately after ecdysis into 4th instar (4-12S, 4-20S), the growth of which was arrested and they eventually died. In contrast to the salivary gland and epidermis, a low level of expression was continuously observed in the brain, aside from the large increase associated with pupation.

## Discussion

### Evolution of BR-C zinc finger sequences

In the present study, seven types of *BR-C* transcripts, which contained one or two of the Z1–Z6 sequences, were identified in *P. hilaris*. The Z1–Z4 sequences are relatively common in insects (Spokony and Restifo 2007). In contrast, the Z5 isoform has only been reported for two hemimetabolous insects, *B. germanica* (Piulachs et al. 2010) and *Frankliniella occidentalis* (Minakuchi et al. 2011), and one holometabolous insect, *Tribolium castaneum* (Konopova and Jindra 2008; Suzuki et al. 2008). Piulachs et al. (2010) suggested that Z5 was lost in the derived groups of holometabola. Our result that the BR-C protein containing the Z5 sequence will not be produced in *P. hilaris* due to the presence of a premature stop codon in the Z5/Z6 isoform appears to be consistent with the loss of Z5 hypothesis.

The Z6 sequence had only been reported for a hemimetabolous insect, *B. germanica* (Piulachs et al. 2010). The survey of public databases conducted in the present study suggested that the Z6 sequence was absent in the genomes of insects whose genomes were sequenced, e.g., *Drosophila*, *Bombyx*, and *Tribolium*. Two scenarios have been suggested for the evolution of Z6 by Piulachs et al. (2010). One is that Z6 evolved in the cockroach lineage after holometabola and hemimetabola split. The other is that Z6 had been present before the split and was lost during the course of the evolution of holometabola. Because the former scenario does not explain the presence of Z6 in *P. hilaris*, a holometabolous species, the results of the present study support the latter scenario. The loss of Z6 in coleopteran species is of particular interest. The absence of Z6 in the genome of *Tribolium* suggests that the loss of Z6 occurred within group(s) of Coleoptera more than once during the course of their evolution.

Taken together, our results suggest that although insects may once have acquired six (Z1–Z6) BR-C isoforms, the number of isoforms being used decreased during the course of evolution, particularly in holometabolous insects. This view is in agreement with the suggestion by Piulachs et al. (2010); however, investigation of the BR-C isoforms in various insects belonging to different orders is necessary to clarify the evolutionary history of the BR-C isoforms in insects.

### Expression profile of *PhBR-C*

An increase in *PhBR-C* transcripts in the brain, salivary gland, and epidermis was observed from the gut purge stage to prepupal stage (Figure 6). In *Drosophila*, *Manduca*, and *Bombyx*, the expression of *BR-C* in many tissues was shown to be under the control of 20-hydroxyecdysone (20E) and JH; being induced by 20E and suppressed by JH (Zhou et al. 1998; Zhou and Riddiford 2002; Reza et al. 2004). However, responsiveness to these hormones appeared to be high in tissues such as the salivary gland, silk gland, epidermis, and muscle, and low in tissues such as the brain and fat bodies. While the expression of *BR-C* in the majority of tissues is specifically induced during metamorphosis, the low, but constant expression of *BR-C* has been observed in the brain and fat bodies (Emery et al. 1994; Ijiro et al. 2004; Nishita and Takiya 2004). BR-C in the brain and fat bodies may have functions not directly related to metamorphosis, and may be useful in studying the transition of *BR-C* functions during the course of the evolution of holometabola. In this regard, BR-C isoforms have been suggested to be involved in the differentiation of neurons in the *Drosophila* brain (Spokony and Restifo 2009; Zhou et al. 2009; Dincer 2011).

When fourth instar larvae were starved after 4 days of feeding (4-4FS), JH titers markedly decreased over the subsequent 24 h and never recovered, and a significant increase in 20E titers was observed on day 6, followed by a large peak on day 11 (Munyiri and Ishikawa 2005a). The increase observed in *PhBR-C* expression levels in the epidermis of 4-4F5S and 4-4F8S larvae (Figure 6) may have been induced by the small surge in 20E titers in the absence of JH. *PhBR-C* expression levels in starved larvae expected to undergo larval molt (3-3F3S) were low. Therefore, starvation *per se* appeared to have no direct effect on the expression of *PhBR-C*.

No significant increase was observed in the expression level of *BR-C* in any tissues of the larvae starved immediately after ecdysis into 4th instar (4-12S, 4-20S), which fell into developmental arrest and eventually died. The absence of the expression of *BR-C* in these larvae suggests that their development was arrested before pupal commitment. The same treatment, starvation, provoked different consequences in larvae with different feeding backgrounds (cf. 4-4F8S and 4-12S). The role of the insulin/TOR signaling system, which senses the nutritional conditions of the body, in regulating growth has been studied extensively using lepidopteran and dipteran model species (Mirth and Riddiford 2007; Nijhout et al. 2014). However, the involvement of the insulin/TOR system in the regulation of metamorphosis in *P. hilaris* has yet to be studied.

## Electronic supplementary material

Additional file 1: Table S1: Description of data: Oligonucleotide primers used for cloning and RACE. (PDF 10 KB)

Additional file 2: Table S2: Description of data: GenBank accession numbers and regions of the nucleic acid sequences used for phylogenetic analyses. (PDF 18 KB)
