# Supplementary material for: Cloning, phylogeny, and expression analysis of the Broad-Complex gene in the longicorn beetle Psacothea hilaris
Source: Springerplus. 2014 Sep 18;3:539. doi: 10.1186/2193-1801-3-539 (PMC4175664; doi:10.1186/2193-1801-3-539)
Supplement: Supplementary file 1 — Additional file 1: Table S1: Description of data: Oligonucleotide primers used for cloning and RACE. (PDF 10 KB) [file 40064_2014_1235_MOESM1_ESM.pdf]

Table 1. Oligonucleotide primers used for cloning and RACE

**Degenerate primers ***	
Br-f1	5'-tagtctagaagcttTTYTGYYTNMGNTGGAAYAA-3'
Br-R1	5'-tgtctgcacgaattcRAARTANGGNGARCANGC-3'
Br-R2	5'-tgtctgcacgaattcRAARTANGGRCTRCANGC-3'
Z2-DG1	5'-CANCGRTAYTCYTCYTGNC-3'
Z3-DG1	5'-AGNGTRTCNGAYTGYTC-3'
Z5-DGRV1	5'-CAGRTGRCGYTGNAGNGACCA-3'
Z5-DGRV2	5'-GAYACNCAYTTYTAYACGCCGCA-3'

**Primers for RACE**	
3'-RACE Primer	5'-CTGTGAATGCTGCGACTACGAT-3'
BR-FW1	5'-TCACATCAGCTTTCGAGAACCTCAGA-3'
BR-FW2	5'-CATTAGCGTGCGATGGAAAGAGC-3'
PW-FW1	5'-AACGAACATAAAAGGGCCAGGTC-3'
PW-FW2	5'-CCGGCAGCTTTAACTTCAGTATGG-3'
Z2-FW1	5'-GTAAGGTGCTGTGCTCGAAAGC-3'
Z2-FW2	5'-CTACCGCACCAAGAACTCACTC-3'
Z2-FW3	5'-GCTTTCAGCTATGCAAGCACAC-3'
Z3-FW1	5'-ATCAACGAGCCACAGGAGTGC-3'
Z3-FW2	5'-AAGACCACGGCCATCAAGAG-3'
Z5-PWFW1	5'-GCCTGGTCTGAGTCCTCTCTAACG-3'
Z5-PWFW2	5'-ACTGTAAAATTTGCG-3'
5'-RACE Primer	5'-GTCTACCAGGCATTCGCTTCAT-3'
BR-RV2	5'-TCTTTCCATCGCACGCTAATGTCA-3'
BR-RV1	5'-TCTGAGGTTCTCGAAAGCTGATGTGA-3'

* The sequences written in lower case include the restriction enzyme recognition site.
