# Supplementary material for: Cloning, phylogeny, and expression analysis of the Broad-Complex gene in the longicorn beetle Psacothea hilaris
Source: Springerplus. 2014 Sep 18;3:539. doi: 10.1186/2193-1801-3-539 (PMC4175664; doi:10.1186/2193-1801-3-539)
Supplement: Supplementary file 2 — Additional file 2: Table S2: Description of data: GenBank accession numbers and regions of the nucleic acid sequences used for phylogenetic analyses. (PDF 18 KB) [file 40064_2014_1235_MOESM2_ESM.pdf]

Table 2. GenBank accession numbers and regions of the nucleic acid sequences used for phylogenetic analyses

Species	Z1		Z2		Z3	
	Accession No.	Region	Accession No.	Region	Accession No.	Region
Acheta domesticus	DQ176003.1	1309-1464				
Acyrthosiphon pisum			XM_001942646.2	2554-2709		
Aedes aegypti	AY499537.1	1568-1723	AY499538.1	1492-1647	AY499539.1	1564-1719
Anopheles gambiae			XM_308027.2	58-213	XM_308028.4	208-363
Apis florea	XM_003699020.1	39-194	XM_003695045.1	22-177	XM_003695045.1	483-328
Apis mellifera	AB208106.1	13-168	XM_003249278.1	117-272	AB208108.1	22-177
Bactrocera dorsalis					JX069964.1	372-527
Blattella germanica	FN651774.1	1367-1522	FN651775.1	1364-1519	FN651776.1	1403-1558
Bombus impatiens			XM_003494365.1	1251-1406	XM_003494366.1	85-240
Bombus terrestris	XM_003400277.1	67-222	XM_003403336.1	69-224	XM_003400164.1	85-240
Bombyx mori	AB166725.1	1199-1354	AB181197.1	1331-1486	AB113089.1	8751-8906
Chrysopa pallens			GAGF01032915.1	52-207	GAGF01032915.1	343-498
Culex quinquefasciatus			XM_001844944.1	157-312	XM_001844945.1	154-309
Dendroctonus ponderosae	APGK01036020.1	10174-10019	APGK01036020.1	917-762	APGK01036019.1	23086-22931
Drosophila melanogaster	X54663.1	2106-2261	X54665.1	1707-1862	X54664.1	2151-2306
Frankliniella occidentalis			AB572561.1	1743-1898	AB572562.1	1692-1847
Manduca sexta			AF032674.1	19-174	AF032675.1	28-183
Megachile rotundata					XM_003704314.1	1066-1221
Nasonia vitripennis	XM_001601980.2	1184-1339	XM_003426018.1	58-213	XM_001601297.2	130-285
Pediculus humanus corporis			XM_002426486.1	1132-1287	XM_002426486.1	1354-1509
Psacothea hilaris	AB857715.1	1192-1347	AB857716.1	1201-1356	AB858991.1	1228-1383
Tribolium castaneum	EU200754.1	22-177	EU200755.1	28-183	EU200756.1	55-210

Table 2. Continued

Species	Z4		Z5		Z6	
	Accession No.	Region	Accession No.	Region	Accession No.	Region
Acyrthosiphon pisum	XM_001942646.2	2136-2291				
Aedes aegypti	XM_001659167.1	1658-1813				
Anopheles gambiae	XM_565666.4	1567-1722				
Apis florea	XR_142997.1	742-897				
Apis mellifera	AB207270.1	1384-1539	NR_039480.1	6-134		
Blattella germanica	FN651777.1	1409-1564	FN651778.1	1388-1543	FN651779.1	1424-1597
Bombus terrestris			XM_003400278.1	2373-2528		
Bombyx mori	AB166727.1	1193-1348				
Chrysopa pallens	GAGF01019727.1	301-146				
Dendroctonus ponderosae	APGK01036020.1	6955-6800				
Drosophila melanogaster	U51585.1	2689-2844				
Frankliniella occidentalis			AB572563.1	1689-1844		
Manduca sexta	AF032676.1	1663-1818				
Psacothea hilaris	AB858992.1	1219-1374	AB858993.1	2480-2635	AB858994.1	1219-1374
Tribolium castaneum	EU200757.1	46-201	NM_001111264.1	1504-1659		
